# Use and pattern of previous care received by infertile Nigerian women

**DOI:** 10.1186/s40738-019-0068-6

**Published:** 2019-12-03

**Authors:** Amina Mohammed-Durosinlorun, Joel Adze, Stephen Bature, Amina Abubakar, Caleb Mohammed, Matthew Taingson, Lydia Airede

**Affiliations:** Department of Obstetrics and Gynaecology, Faculty of Medicine, Kaduna State University, Kaduna, Nigeria

**Keywords:** Pattern, Previous fertility treatment, Infertility, Nigeria

## Abstract

**Background:**

Prevalence of infertility in sub-Saharan Africa is high yet fertility care, its development and access is limited in resource-poor countries like Nigeria so infertile women resort to different forms of treatment. This study aimed to determine the use and pattern of previous treatments.

**Methodology:**

This was a descriptive Cross Sectional study conducted at a tertiary hospital in North-Western Nigeria. Interviewer administered pretested questionnaires were administered to 236 consenting clients seen at their first visit to the gynaecology clinic with complaints of inability to conceive, between January 2016 to March 2018. We collected information on demographic and reproductive characteristics, previous fertility treatment and other data relevant to infertility. Descriptive analysis was done using SPSS software version 22.

**Results:**

Two hundred and thirty six clients participated in the study and majority were 20–29 years (44.5%), with a mean age of 31.5 ± 7.4, while the mean age of their husbands was 41 ± 8.0. More clients were educated up to secondary level or above (80.9%), with more Muslims (65%) than Christians. All clients were married except one, most clients had been married for 5 years or more, 18.2% were in their second order of marriage and 28% were in polygamous marriages. Many of the clients were homemakers (46.6%) and earned an average monthly income of less than fifty thousand naira. About 59.3% of clients presented with primary infertility, with 15.7% being infertile for duration of more than 10 years. One hundred and forty six respondents (61.9%) had received previous hospital treatments before presentation to our facility, 37% had visited more than three hospitals, 70% did not have adequate investigations done, treatment was successful in 15% while 40.7% received traditional treatments. Husbands of women receiving previous treatment were slightly older (*p* value < 0.05).

**Conclusion:**

Majority of woman have multiple and unnecessary visits to several hospitals for infertility care with little positive results despite time and resources spent. Quality of infertility care needs to be improved.

## Background

Infertility is prevalent worldwide affecting about 5–8% of couples [1]. Prevalence of infertility in sub-Saharan Africa is higher, with 10–30% of couples affected in Nigeria [2]. It is one of the commonest reasons for women to seek gynaecologic consultation [3–5]. Its aetiology in Nigeria was found to be mainly related to post infectious causes; sexually transmitted infections, post abortal and puerperal sepsis [6].

Being able to get pregnant is a big part of the marriage institution, especially in the African cultural context. Hence infertility is associated with a lot of negative psychosocial and other consequences such as stigma, deprivation and neglect, violence, marital problems and mental health issues [1, 7].

Despite this large burden, very few infertility-management programs exist [5]. Fertility care, its development and access is limited in resource-poor countries [8] like Nigeria, overshadowed by competing and more important reproductive health issues [9] like high maternal mortality rates. Most times the burden of infertility lies squarely on the couple alone, with a greater burden on the woman.

To satisfy their needs and end their suffering, infertile women may resort to different forms of treatment. This study aimed to determine what prior treatments have been received, and if infertility care was adequate. Very few studies if any, have looked into this aspect of infertility care in the study setting. One Nigerian study by Ola et al. [10] noted that most women resort to faith healing first to treat infertility, but about half of them still complement it with formal treatment, which was not specified in the study.

Findings of this study will contribute to literature on the pattern and quality, or lack thereof, of infertility services women access in low resource settings. It will also have implications on recommendations to improve infertility assessment and management.

## Methodology

Ethical approval for the study was received from the Barau Dikko Teaching Hospital (BDTH) Health and Research Ethics Committee and verbal informed consent obtained from participants.

This was a descriptive cross sectional study done between January 2016 to March 2018.

The study was conducted at the BDTH, Kaduna, North-Western Nigeria. The hospital serves as a major referral facility for the metropolis and its environs. The gynaecology clinic is run twice a week with an average number of 50 new clients seen weekly, half of which are infertility clients.

The study participants were women presenting for the first time to the gynaecology clinic with complaints of inability to conceive, and consented to participate in the study. Women were eligible to participate irrespective of their age or duration of complaint.

The minimum sample size was determined using the formula by Lemeshow et al. [11], and prevalence rate for infertility of 15.7% [12]. The study was part of a larger study on infertility at the study location. Convenient sampling was done and interviewer administered pretested questionnaires were administered to 236 consecutive consenting clients. Questionnaire was developed and face validation done by authors. It was pretested on twenty infertile women and questions rephrased where required. Questions were semistructured with both open and close ended questions. Information was gotten on demographic and reproductive characteristics, previous fertility treatment and other data relevant to infertility. Information of previous fertility treatment included number and type of hospital where treatment was received, type of investigations done to participant and partner, and if traditional treatment was used and type, and if treatment was successful. Successful treatment was defined as the client becoming pregnant and was confirmed by a pregnancy test or ultrasound, irrespective of outcome (miscarriage or live birth). Because it is a low resource setting, investigations done were deemed “adequate” if at least four tests were done to assess both female and male factor infertility. These include a semen analysis (male factor) and a high vaginal swab, pelvic ultrasound and hysterosalpingogram (female factor). If one or more of the four investigations were not done, it was deemed “inadequate”. It was sometimes necessary for interviewer (attending gynaecologist) to cite results of previous investigations done if available, or explain the investigation or treatment being asked for.

Descriptive analysis with frequencies and percentages was done using SPSS computer software version 22. Chi square was used to compare proportions between groups and a *p* value of < 0.05 was considered to be statistically significant.

## Results

### Demographic and general characteristics of respondents

As shown in Table 1, two hundred and thirty six clients participated in the study and majority were 20–29 years (44.5%), with a mean age of 31.5 ± 7.4 years, minimum age of 17 years and maximum age of 51 years. Majority of their husbands (41.5%) were aged between 30 and 39 years, with a mean age of 41 years ±8.0, a minimum age of 25 years and maximum age of 65 years. More clients were educated up to secondary level or above (80.9%), there were slightly more Muslims (65%) than Christians. All clients were married except one, most clients had been married for 5 years or more, 18.2% were in their second order of marriage and 28% were in polygamous marriages. Many of the clients were homemakers (46.6%) and majority earned an average monthly income of less than fifty thousand naira. About 59.3% of clients presented with primary infertility, with 15.7% being infertile for duration of more than 10 years. Sociodemographic characteristics were similar among women that received previous treatments or not but, Husbands of women receiving previous treatment were slightly older (*p* value < 0.05).

**Table 1:** Demographic and general characteristics of respondents

Characteristics	Women with no previous treatment frequency (%)	Women with previous treatment frequency (%)	Total population Frequency (%)	Test statistics (df = degree of freedom)
Clients age				
< 20	1 (100)	0 (0.0)	1 (0.4)	Likelihood ratio 9.026 df 4 p value 0.060
20–29	48 (45.7)	57 (54.3)	105 (44.5)	
30–39	32 (34.8)	60 (65.2)	92 (39.0)	
40–49	9 (29.0)	22 (71.0)	31 (15.7)	
> 50	0 (0.0)	1 (100)	1 (0.4)	
Total	90 (38.0)	146 (62.0)	236 (100)	
Husband’s age				
< 20	0 (0.0)	0 (0.0)	0 (0.0)	Pearson Chi Square 11.324 df 3 p value 0.01
20–29	8 (80%)	2 (20)	10 (4.2)	
30–39	40 (40.8)	58 (59.2)	98 (41.5)	
40–49	31 (37.3)	52 (62.7)	83 (35.2)	
> 50	11 (24.4)	34 (75.6)	45 (19.1)	
Educational level				
None	1 (33.3)	2 (66.7)	3 (1.3)	Pearson Chi Square 2.740 df 4 p value 0.602
Quaranic only	7 (41.2)	10 (58.8)	17 (7.2)	
Primary	9 (36.0)	16 (64.0)	25 (10.6)	
Secondary	44 (43.6)	57 (56.4)	101 (42.8)	
Tertiary	29 (32.2)	61 (67.8)	90 (38.1)	
Marital status				
Married	89 (37.9)	146 (62.1)	235 (99.6)	Pearson Chi Square 1.629 df 1 p value 2.02
Single	1 (100)	0 (0.0)	1 (0.4)	
Duration of marriage				
≤ 1 year	14 (51.9)	13 (48.1)	27 (11.4)	Pearson Chi Square 2.982 df 3 p value 0.394
> 1-5 years	32 (34)	62 (66)	94 (39.8)	
> 5-10 years	24 (40)	36 (60)	60 (25.4)	
> 10 years	20 (36.4)	35 (63.6)	55 (23.3)	
Order of marriage				
First	75 (38.9)	118 (61.1)	193 (81.8)	Pearson Chi Square 0.236 df 1 p value 0.627
Second or more	15 (34.9)	28 (65.1)	43 (18.2)	
Type of marriage				
Monogamy	67 (39.4)	103 (60.6)	170 (72.0)	Pearson Chi Square 0.420 df 1 p value 0. 517
Polygamy	23 (34.8)	43 (65.2)	66 (28.0)	
Occupation				
Student	9 (60)	6 (40)	15 (6.4)	Pearson Chi Square 9.424 df 4 p value 0.051
None/ Home maker	48 (43.6)	52 (56.4)	110 (46.6)	
Self employed	20 (33.3)	40 (66.7)	60 (25.4)	
Privately employed	7 (33.3)	14 (66.7)	21 (8.9)	
Civil servant	6 (20)	24 (80)	30 (12.7)	
Average monthly income in Naira				
< N10,000	50 (44.2)	63 (55.8)	113 (48.0)	Pearson Chi Square 6.985 df 3 p value 0.072
N10,000 - < N50,000	34 (37.4)	57 (62.6)	91 (38.5)	
N50,000 - < N100,000	5 (20)	20 (80)	25 (10.5)	
> N100,000	1 (14.3)	6 (85.7)	7 (3.0)	
Type of infertility				
Primary	33 (34.4)	63 (65.6)	96 (40.7)	Pearson Chi Square 0.970 df 1 p value 0.325
Secondary	57 (40.7)	83 (59.3)	140 (59.3)	
Duration of fertility				
≤ 1 year	15 (60)	10 (40)	25 (10.6)	Pearson Chi Square 11.324 df 3 p value 0.01
> 1 year to 5 years	46 (37.7)	76 (62.3)	122 (51.7)	
> 5 year to 10 years	19 (36.5)	33 (63.5)	52 (22.0)	
> 10 year	10 (27)		37 (15.7)	
Parity				
0	49 (34.8)	92 (65.2)	141 (59.7)	Pearson Chi Square 1.827 df 2 p value 0.401
1–4	40 (43.5)	52 (56.5)	92 (39.0)	
> 4	1 (33.3)	2 (66.7)	3 (1.3)	
Total	90 (38.0)	146 (62.0)	236 (100)	

### Previous treatments

**Fig. 1:**
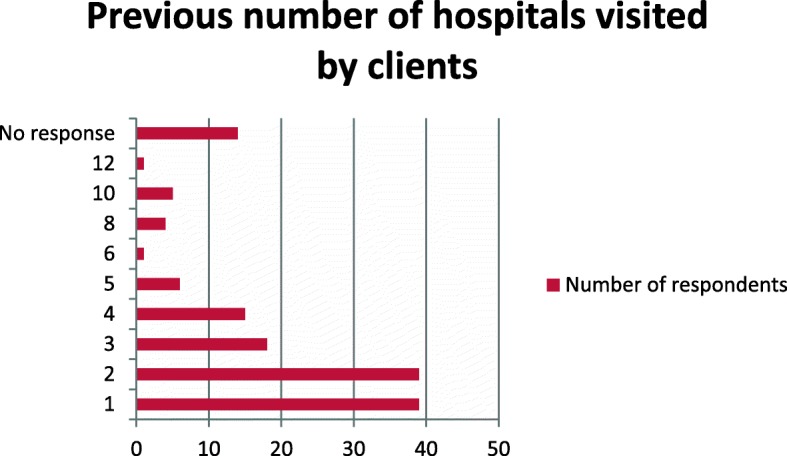
Number of previous hospitals visited

One hundred and forty six respondents (61.9%) had received previous hospital treatments before presentation to our facility. Only ninety respondents responded on where they received such treatments, mostly from both public and private hospitals, and 37% had visited more than three hospitals (Tables 2 and 3, Fig. 1).

**Table 2:** Characteristics of previous hospital treatments recieved

Previous treatment variable	Frequency (total = 146)	% (total = 100)
Where previous treatment was gotten		
Private hospital	44	30.1
Public hospital	31	21.3
Both	61	41.8
No response	10	6.8
Average number of hospitals previously visited		
1	39	26.7
2	39	26.7
≥ 3	54	37.0
No response	14	9.6
Previous investigations done		
Adequate	37	25.3
Inadequate	102	69.9
No response	7	4.8
Previous treatment successful		
Yes	22	15.1
No	124	84.9
Average amount spent in previous hospitals		
< N10,000	15	10.3
N10,000 - < N50,000	46	31.5
N50,000 - < N100,000	30	20.5
> N100,000	28	19.2
No response	27	18.5

**Table 3:** Summary of types of previous treatments received

Traditional treatment	Conventional treatment
*Use of traditional treatment*	*Use of conventional treatment*
No 140 (59.3%)	No 90 (38.1%)
Yes 96 (40.7%)	Yes 146 (61.9%)
*Types of trado-medicine*	*Medical*
Islamic medicine - 2	Unspecified-146
Moringa seeds-1	Clomiphene citrate-10
Oral and Vaginal herbs −2	Antibiotics-5
Oral herbs − 84	Bromocriptine- 2
Oral herbs and Garlic- 1	Hormonal-2
Oral herbs and Islamic medicine-2	*Surgical*
Oral herbs and prayers-1	Hydrotubation-6
Unspecified-1	Myomectomy-1
Vaginal herbs-1	Manual vacuum aspiration-1
Vaginal soap −1	*In vitro fertilization-3*

Investigations were generally considered adequate if a minimum of genital infection screening, pelvic ultrasonography and tubal patency testing was done in the female partner and semen analysis was done in the male partner. About 70% of clients did not have all these basic tests done. Only two clients had a diagnostic laparoscopy done, and a few were able to do hormonal assays when indicated.

Any previous history of hospital treatment was successful in only 15% of cases. All women had received some form of medical treatment but most were unable to specify them. For those women who were able to specify, treatment consisted of antibiotics, ovulation induction with clomiphene citrate, bromocriptine and other unspecified medications as shown in Table 3. In one case, fertility was achieved after a myomectomy. Six women had hydrotubation, while three respondents had attempted in vitro fertilization which failed.

Apart from hospital treatments, 96 clients had also received traditional treatments consisting of mainly oral herbs ground to a powder or mixed in local cereals. The oral herbs are used alone, or with other methods as shown in Tables 3 and 4. Any previous history of traditional treatment was associated with 8.3% success, but only when it was combined as shown in Table 4.

**Table 4:** Comparing success and type of previous treatment

Type of treatment	Was previous treatment successful?			Test statistics
	No (%)	Yes (%)	Total (%)	
Any previous history of hospital treatment				Pearson’s Chi square 14.956 df 1 p value 0.000
No	90 (100%)	0 (0.0%)	90 (100%)	
Yes	124 (84.9%)	22 (15.1%)	146 (100%)	
Any previous history of traditional treatment				Pearson’s Chi square 0.187 df 1 p value 0.665
No	126 (90.0%)	14 (10.0%)	140 (100%)	
Yes	88 (97.1%)	8 (8.3%)	96 (100%)	
Combined and/or individual treatments				
Both	68 (89.5%)	8 (10.5%)	76 (100%)	Pearson’s Chi square 18.825 df 3 p value 0.000
Only hospital treatment	56 (80%)	14 (20%)	70 (100%)	
Only traditional treatment	20 (100%)	0 (0.0%)	20 (100%)	
No previous treatment	70 (100.0%)	0 (0.0%)	70 (100%)	
Total	214 (90.7%)	22 (9.3%)	236 (100%)	

## Discussion

The predominant perception is that infertility may not be an important issue for African countries, as compared to other parts of the world. This study however presents a different picture and highlights one of the problems faced by infertile women in Nigeria. The study presents findings of a cross sectional survey administered to women with infertility, to understand the pattern and use of fertility services prior to contact with the at the study centre.

About 62% of respondents had received previous hospital treatments before presentation to our facility. This is slightly higher than what was found in Iran (59.2% of 118 couples) [13].

The mean age of the sample population was 31.5 years, while their husbands had a mean age of 41 years. It is importance that any previous or current infertility care received is of good quality as time is of essence and fertility is known to decline with increasing age. The age factor is important, as the day-specific probability of conception declines with age. Dunson et al. [14] found that women’s fertility begins to decline in the late 20s, with a substantial decrease by the late 30s, and that also, when other factors are corrected for, there is a decrease in male fertility from 30 years of age onwards. ART treatment success also varies with age, and the chance of a live birth is significantly decreased after 35 years of age and is < 10% after the age of 40 years [15]. Husbands of women receiving previous treatment were slightly older than those that did not receive previous treatments, and the difference was statistically significant. While the exact reason for this is unclear, it may reflect the delay spent in seeking previous care.

Less than half of clients had been married for 5 years or more, and 38% have been infertile for duration of 5 or more years. This means they may have been exposed to the psychological adverse effect of infertility for long periods [16]. If women had received previous treatment in facilities where quality is good and resources available, then fertility treatment, should take a much shorter period and reduce the suffering women are exposed to.

Respondents visited both public and private hospitals. Public hospitals are more affordable, and public tertiary centres are perceived to have better trained personnel but have long waiting times. However, more private centres provide artificial reproductive techniques (ART) should it be required than the Nigerian public sector.

About 37% had visited more than three hospitals. This is similar to other studies in Indonesia and Iran where the mean number of specialists visited for fertility consultation was three [13, 17], for a range of reasons including failed treatment, a more reputable and accommodating doctor among others. It is not unusual for clients to seek second opinions about their diagnosis, especially if there is initial denial of infertility. However, visiting three or more may be more time and resource wasting. ART subspecialists prefer early referrals. Referrals of patients to specialists are affected by a range of factors, including characteristics of the referring physicians, the patients and the particular type of specialist [18]. Internationally, reproductive tourism to other countries is mostly due to legal restrictions placed on certain forms of treatment in the host country [19].

Basic investigations for infertility should assess both male and female factors. A high proportion of respondents (70%) that had previous hospital treatments did not have these basic tests done. The quality of fertility care and completeness of investigation may depend on the type, level and experience of health worker seen, but this was not fully explored in our study. One study in Indonesia [17] found that though women preferred to see specialists/gynaecologists, they also visited general practitioners, nurses and midwives for fertility concerns. Infertility is usually wrongly regarded as female problem in this environment [20] so it is sometimes difficult to get the male partner to bring a semen sample for analysis, more so if he has more than one wife or other children; though who is to say these children are actually his? Infertile women have been known to cope with infertility by infidelity [21].

Omitting tests is sometimes done to save costs but it is no excuse for missing out basic tests. Laparoscopy for example can be omitted from the infertility work-up to save cost when the hysterosalpingography is normal and there is no abnormal contributing history [22], and it is not widely available in our setting. The indirect method to assess male semen parameters (postcoital test) would have been ideal in this setting [23] but there may still be logistic hurdles to overcome; transport, training and lab preparedness.

The FIGO Fertility Tool Box [24] simplifies infertility care by providing tools on a whole range of issues; from overcoming barriers, to prevention, treatment and referral. It is based on the best available evidence, comprehensive, integrated and easy to use, especially in low-resource settings and by mid-level practitioners. Its use may likely improve the quality of fertility care offered.

Previous hospital treatment was successful in only 15% of cases. This is similar to another Nigerian study [25]. This is low, probably due to the limited scope available, especially as proper diagnosis may have been lacking. Since the commonest cause of infertility in this setting is infection [6] likely resulting in tubal damage, ART may be appropriate in some cases [25]. The use of antibiotics while curing on-going sepsis, will not improve tubal damage. Only three women had attempted ART which failed. Training and availability of ART, which is useful not only for male factor infertility, but also unexplained infertility is poor in our setting, and even when available, is restricted by financial access. ART is not currently available in our centre so we refer to the private centres.

The commonest treatment offered was ovulation induction with clomiphene citrate. Women already know this drug from multiple consultations, and abuse it/ self-medicate despite potential serious side effects [26].

Only six women had hydrotubation in our study. The procedure for hydrotubation is not standardized and usually involves an attempt to flush a large amount of fluids trancervically (with or without sedation, antibiotics or steroids) in the hope that it might correct some tubal blockage. The manner it was done in these women could not be ascertained. Though hydotubation is discouraged by ART specialists, its use is actually more widespread in Nigeria than reported, because women are unable to afford ART. There are reports that with careful selection, hydrotubation may be useful in resource poor countries, especially in patients with incomplete tubal occlusion [27].

Apart from hospital treatments, 40.7% of respondents also received traditional treatments. This is similar to a study in Freetown, where 36.5% of 167 women used herbal medicine for infertility treatment [8]. Another Nigerian study reported that more than two-thirds of infertile couples (69%) seek care from a traditional complementary medicine practitioner [27]. High cost of conventional therapy, cultural and religious beliefs, and societal pressure to conceive may influence a woman’s decision to seek traditional treatment [8]. Another reason couples may opt for traditional assistance is to avoid detection of a male factor and accompanying social blame [2, 28]. The fact that health-seekers often seek health care (conventional and traditional) concurrently and sequentially was earlier confirmed by others [2, 29, 30].This study however showed that success was higher with those that received conventional hospital treatment than those receiving traditional treatment. Those receiving only previous hospital treatment also had higher success than those who received combine care. This is not surprising as dosage, drug interactions etc. of herbs are difficult to ascertain so sometimes they may cause more harm than good.

### Study limitations

This study was a questionnaire based survey so it is subject to significant recall bias. Some bias may also have been introduced because women with unsuccessful treatments are more likely to have visited the clinic than those with successful treatment who may have no need to visit the clinic.

The study did not delve into great details of some information which would also have been useful e.g. causes of male infertility and treatments, specific types of herbs used by women and their ingredients and reasons why women visit multiple hospitals.

Overall the study raises a lot of interesting observations but is limited by its small size and hospital setting which may make conclusions difficult to extrapolate to the whole population. A qualitative method may have explored reasons for multiple visits more deeply. It would also have been interesting to note the situation at different levels of care, and between rural and urban milieu, which was not done in this particular study.

## Conclusion

Sociodemographic characteristics were similar among women that received previous treatments or not but, Husbands of women receiving previous treatment were slightly older (*p* value < 0.05). An emerging pattern seen is that majority of women studied have multiple and unnecessary visits to several hospitals for infertility care, which may be of low quality since inadequate investigations were done. It is therefore not surprising that there are little positive results despite time and resources spent. This may also lead to delays in accessing ART and prospects of reduced success with increasing age. A lot of women still resort to the use of traditional medicines, mainly herbs which were not as effective as conventional treatment. Vast herbal resources remain unexplored and studies need to be conducted to see if they have any potential for infertility treatment, and to ensure proper regulation, safety and non-exploitation of desperate women. Most importantly quality of infertility care needs to be improved by better education of women, training of health workers, early referrals to fertility and ART specialists as required, and innovative funding options to widen scope of fertility care offered, and access to it. There should be wider dissemination of the FIGO fertility tool box, customised to context and used at all levels of care.

## Data Availability

The datasets used and/or analysed during the current study are available from the corresponding author on reasonable request.

## Abbreviations

ART: Artificial Reproductive Technology
BDTH: Barau Dikko Teaching Hospital
df: degree of freedom
FIGO: International Federation of Gynaecologists and Obstetricians
SPSS: Statistical Package for Social Sciences
